# Development of a Machine Learning Algorithm for Differential Diagnosis Between Primary Immune Thrombocytopenia and Connective Tissue Disease‐Related Thrombocytopenia in Pediatric Patients

**DOI:** 10.1002/jcla.70337

**Published:** 2026-09-03

**Authors:** Furong Kang, Mei Yan, Yingbin Yue, Xuemei Wang, Yu Liu

**Affiliations:** ^1^ Department of Pediatric Center The First Affiliated Hospital of Xinjiang Medical University Urumqi Xinjiang People's Republic of China

**Keywords:** children, connective tissue disease, diagnostic model, immune thrombocytopenia, machine learning

## Abstract

**Background:**

To develop a machine learning model for early differentiation of primary immune thrombocytopenia (pITP) from connective tissue disease‐related thrombocytopenia (CTD‐TP) in children presenting with thrombocytopenia.

**Method:**

A retrospective study was conducted on 387 newly diagnosed children with thrombocytopenia. All patients were clinically diagnosed and divided into a training set and a test set in a 7:3 ratio. Six machine learning algorithms, including XGboost, RF, SVM, LR, GBDT, BPNN, were used to establish differential diagnostic models for pITP and CTD‐TP. The model performance was evaluated by accuracy, precision, recall, F1 score, and area under the receiver operating characteristic curve (AUC). Explain the importance and contribution of model feature variables through the Shapley Additive Explanations (SHAP) method.

**Result:**

A total of 387 patients were included in the study, including 347 cases of pITP and 40 cases of CTD‐TP. Based on the above evaluation indicators, the BPNN model had the best performance, with an F1 score of 0.95, an accuracy rate of 94.87%, and an AUC of 0.9738. According to the SHAP value, the top 10 influencing factors were selected as age, erythrocyte sedimentation rate, antinuclear antibodies, IgM, Complement C3, T + B + NK%, B cells, body weight, complement C4, lymphocyte count.

**Conclusion:**

The successful construction of the pITP and CTD‐TP identification models shows that BPNN has the best performance and can provide clinical strategies for early disease identification based on the importance of its variables.

## Graphical abstract

Primary immune thrombocytopenia (ITP) and connective tissue disease‐related thrombocytopenia (CTD‐TP) share overlapping initial presentations in children, often leading to delayed diagnosis and suboptimal management. While existing literature focuses on therapeutic strategies, this study is the first to develop a machine learning (ML)‐based diagnostic model for early differentiation between ITP and CTD‐TP in pediatric patients. Our work leverages clinical and laboratory data from 387 children to create an accessible, high‐performance tool, filling a void in evidence‐based diagnostic approaches for this population.

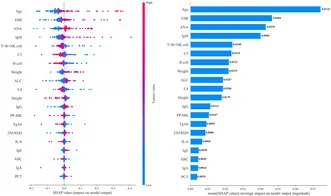

## 1 Introduction

Immune Thrombocytopenia (ITP) is an immune‐mediated disease that destroys platelets, mainly characterized by a low platelet count [1]. Thrombocytopenia usually presents with varying degrees of bleeding symptoms, ranging from skin bruises and purpura to more severe mucosal bleeding, including nosebleeds, gum bleeding, and gastrointestinal bleeding [2]. Beyond the direct risk of bleeding, patients often experience significant fatigue, which is considered a major reason for the reduced health‐related quality of life (HRQoL) in both children and adults with thrombocytopenia [3, 4]. The psychological burden of chronic illness, combined with physical limitations due to bleeding risk, highlights the importance of timely and accurate diagnosis to guide appropriate treatment and reduce long‐term complications [5]. Depending on the cause, ITP can be further divided into primary ITP (pITP) and secondary ITP (sITP). pITP is a diagnosis of exclusion that requires carefully ruling out other potential cases of sITP. sITP results from multiple complex factors or diseases, such as autoimmune diseases, autoimmune thyroid diseases (AITD), lymphoproliferative disorders, or chronic infections. The reported frequency of sITP in children with thrombocytopenia varies across populations and diagnostic criteria; studies in Western countries show a lower prevalence of sITP, accounting for only 20% of cases, whereas research from Asian countries reports a higher prevalence (43%–64%) [6]. Among these, Connective Tissue Disease‐Related Thrombocytopenia (CTD‐TP) is the most common type of sITP, including conditions like sicca syndrome (SS) and systemic lupus erythematosus (SLE) [7]. Connective tissue diseases are a group of autoimmune disorders with diverse clinical manifestations, and a subgroup of patients may first present with isolated thrombocytopenia before developing other clinical symptoms and signs [8]. Therefore, patients with CTD who initially present with low platelet counts often resemble early‐stage pITP, making them hard to distinguish, which can delay diagnosis and treatment. Moreover, the severity of thrombocytopenia often correlates with disease activity and prognosis, so early and accurate differential diagnosis is clinically significant [9].

The treatment strategies, duration, response, and long‐term prognosis of pITP and CTD‐TP are quite different. CTD is essentially a systemic disease that affects multiple organs, so its treatment needs to be based on an overall assessment of the condition, often requiring a combination of immunosuppressants or biologics, with long‐term follow‐up [10]. Patients with ANA‐positive thrombocytopenia may respond well to rituximab (a B cell‐depleting agent targeting cells that produce autoantibodies), while splenectomy has been shown to be more effective in ANA‐negative ITP patients, suggesting different underlying pathophysiological mechanisms [11]. So, building reliable diagnostic prediction models is clinically important for early diagnosis, early treatment, and better long‐term outcomes. Currently, most studies focus only on treatment, and there are no reports yet of large‐scale diagnostic models based on initial clinical features in children [12].

Nowadays, clinical research has entered the era of big data, and more and more computer and data processing technologies are being applied in the medical field [13]. Machine learning (ML), as a continuously developing branch of artificial intelligence, has attracted widespread attention across multiple disciplines. It combines principles from probability theory, statistics, approximation theory, and convex analysis. ML algorithms are good at automatically identifying hidden patterns in datasets and generating predictive insights for unseen data [14]. ML algorithms are a type of algorithm that can automatically discover rules from data and use these rules to make predictions on unknown data. Compared to traditional statistical methods, ML approaches are more flexible in selecting predictive variables and transforming algorithms, and ML has greater parameter capacity, allowing it to learn and fit various clinical qualitative or quantitative data through computers [15]. With the development of ML algorithms in medical research, ML models have been shown to uncover potential associations in clinical features and have already been used for diagnosis of some diseases with good results [16]. In hematology, ML and AI are being increasingly used to tackle complex clinical challenges, including diagnosis and classification of various blood diseases, prognosis evaluation, and prediction of treatment response; the classification accuracy of peripheral blood smears exceeds 95%, and the precise recall of cytological feature detection exceeds 94%. Deep learning architectures have been successfully applied in bone marrow cell classification, differentiation between MDS and ALL, and mutation prediction [17]. However, these methods have not yet been systematically applied to distinguish ITP and CTD‐TP in pediatric populations.

In this study, we conducted a retrospective analysis of 387 patients with thrombocytopenia (347 cases of pITP and 40 cases of CTD‐TP) admitted to our hospital over the past 7 years. We explored potential predictive factors based on simple clinical characteristics at the time of initial diagnosis. By applying ML algorithms, we aim to establish an easy‐to‐use diagnostic model for preliminary differential diagnosis between the two conditions.

## 2 Data and Methods

### 2.1 Research Object

Retrospectively collect the clinical data of children whose first documented clinical manifestation was thrombocytopenia at the First Affiliated Hospital of Xinjiang Medical University from January 2017 to January 2024. Inclusion criteria: (1) Children who had their initial diagnosis at our hospital, aged under 18 years and older than 3 months. (2) Children with pITP whose diagnosis meets the relevant diagnostic criteria in the “Guidelines for the Diagnosis and Treatment of Primary Immune Thrombocytopenia in Children (2019 edition)” [18]. (3) Children whose first clinical symptom was thrombocytopenia (peripheral blood platelets < 100 × 10^9^/L) and were later definitively diagnosed with connective tissue disease. (4) Complete clinical data available. Exclusion criteria: (1) Thrombocytopenia caused by congenital (familial) thrombocytopenia, drug‐induced thrombocytopenia, bone marrow failure, malignancy, etc. (2) Patients with complicated conditions, combined with severe infection or other major organ diseases, such as diabetes, viral hepatitis, etc. (3) Children who visited other hospitals before the initial diagnosis in our hospital and received related treatment. After excluding duplicate cases, a total of 347 cases of pITP and 40 cases of CTD‐TP were ultimately selected. The CTD‐TP group included 20 cases of SLE, six cases of SS, four cases of mixed connective tissue disease (MCTD), and 10 cases of undifferentiated connective tissue disease (UCTD). This study was approved by the Ethics Committee of the First Affiliated Hospital of Xinjiang Medical University (Approval number 241008‐01).

### 2.2 Data Collection and Feature Selection

To achieve early diagnosis, this study only used clinical records from the first visit before any treatment, obtained from electronic medical records. A total of 49 potential feature indicators were identified, covering basic clinical information and laboratory blood test indicators, as shown in Tables 2 and 3. Missing values were estimated using the mean and median based on the distribution of each variable: for normally distributed continuous variables, missing values were replaced with the mean; for non‐normally distributed variables, the median was used. Outliers were identified using the interquartile range (IQR) method and replaced with the median to minimize their impact on model training. When a value does not exist in the dataset, it can be treated as null. Figure 1 shows the percentage of nulls across all attributes. This imputation strategy is reasonable because most variables have a relatively low proportion of missing data. As shown in similar machine learning studies using retrospective clinical data, mean and median imputation are widely accepted methods, which help preserve sample size while minimizing bias. Particularly when the proportion of missing data is low and the missing‐at‐random (MAR) assumption is reasonable, replacing outliers and non‐normally distributed variables with the median can reduce the impact of extreme values while maintaining the overall data distribution; this approach is consistent with established practices in clinical machine learning studies. Mutual information was used to screen feature variables. Mutual information can capture nonlinear relationships and is suitable for both categorical and continuous variables, without assuming any data distribution, thereby reducing the risk of overfitting.

**FIGURE 1:**
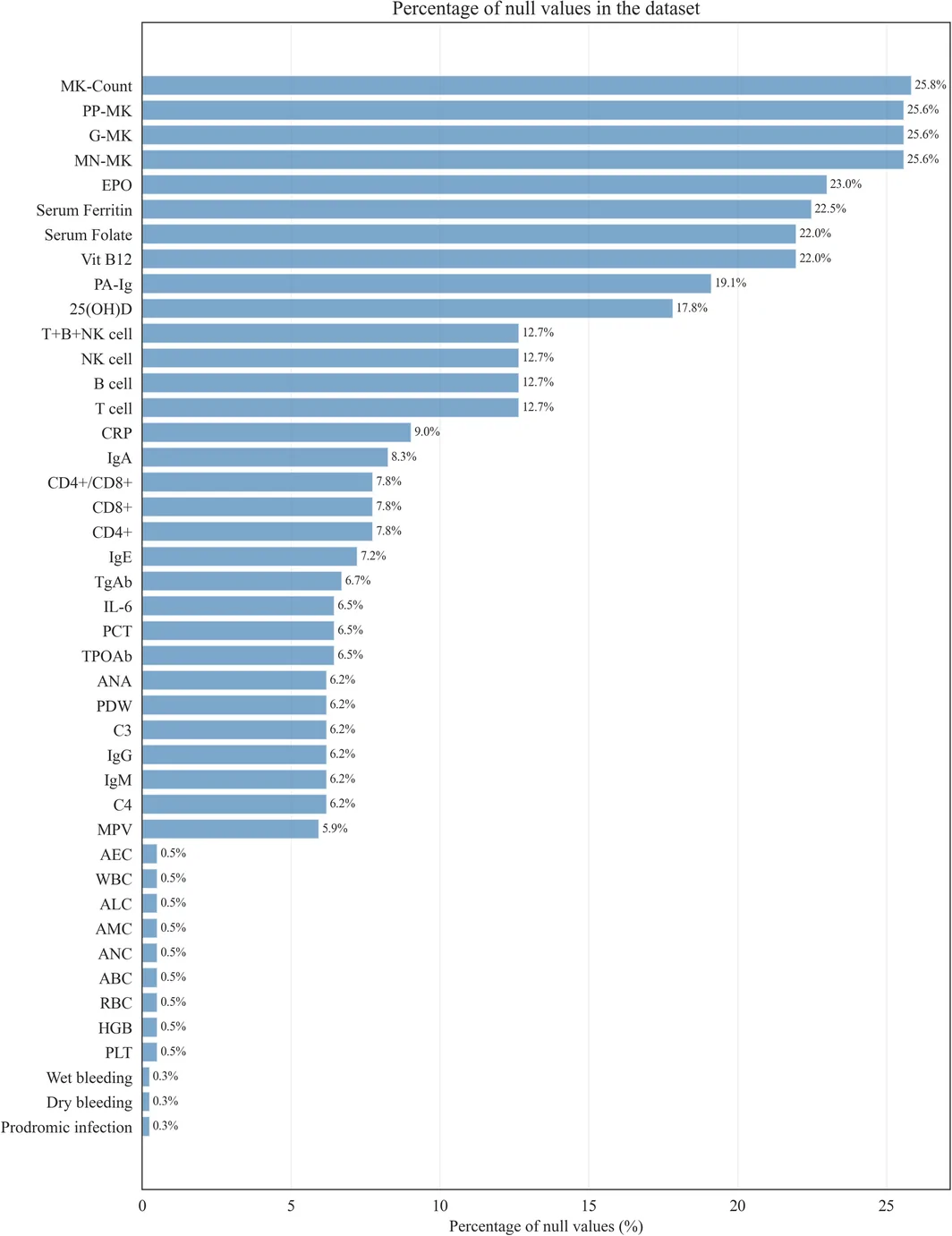
Percentage of empty values.

### 2.3 Machine Learning Model Construction and Performance Evaluation

Using Python 3.10 software as the model development tool, these patients were randomly divided into training and testing sets in a 7:3 ratio. Based on the training set, six classic ML algorithms were used to construct diagnostic models, including extreme gradient boosting (XGboost), random forest (RF), support vector machine (SVM), logistic regression (LR), gradient boosting decision tree (GBDT), and back propagation neural network (BPNN). Some ML algorithms were used to train the models, and grid search methods were used to determine the optimal hyperparameters for each model to avoid overfitting. The hyperparameters used in the models are shown in Table 1.

**TABLE 1:** Hyperparameters used in each model.

Model	Best_Parameters
XGBoost	{‘learning_rate’: 0.1, ‘max_depth’: 3, ‘n_estimators’: 100}
Random forest	{‘max_depth’: 10, ‘min_samples_split’: 2, ‘n_estimators’: 50}
SVM	{‘C': 1, ‘gamma’: ‘scale’, ‘kernel’: ‘sigmoid’}
Logistic regression	{‘C': 10, ‘penalty’: ‘l2’, ‘solver’: ‘saga’}
GBDT	{‘learning_rate’: 0.2, ‘max_depth’: 3, ‘n_estimators’: 200}
BP neural network	{‘alpha’: 0.1, ‘hidden_layer_sizes’: 150, ‘learning_rate_init’: 0.01}

After training, the test set data is predicted, and the performance of the model is evaluated using accuracy, precision, recall, F1 score, and area under the receiver operating characteristic curve (AUC). Among them, Accuracy rate = TP + TN/(TP + TN + FP + FN), Accuracy rate = TP/(TP + FP), Recall rate = TP/(TP + FN), F1 score = 2 × PR × RR/(PR + RR). In order to further analyze the contribution of each feature variable in the model to the prediction results, the Shapley Additive Explanations (SHAP) method was used to explain the constructed machine learning model.

95% confidence intervals (CIs) for accuracy, precision, and recall were estimated using the normal approximation method, where SE = √[*p*(1−*p*)/*n*] and 95% CI = *p* ± 1.96 × SE. For the F1 score, CIs were calculated analogously using the F1 value as the proportion estimate. This approach provides a practical approximation of performance stability given the test set size (*n* = 116).

## 3 Results

### 3.1 Descriptive Statistics of Baseline Data for Research Subjects

Descriptive statistics were performed on the collected baseline clinical data. As shown in Tables 2 and 3 describes several statistical measures for the 49 variables, including mean, median, standard deviation, interquartile range, and percentile of attributes.

**TABLE 2:** Baseline measurement data description.

Attribute	Group	Mean	Median	SD	25th	75th
Age (years)	1	5.26	4.73	3.6612	2.14	7.58
	2	10.15	10.11	3.4479	8.00	13.00
Height (cm)	1	110	110	27.5004	90	129
	2	139	145	20.2960	127	152
Weight (kg)	1	21.8	19.0	12.7899	12.5	26.5
	2	37.3	37.9	15.5913	22.9	45.2
BMI	1	17	16	2.6273	15	18
	2	19	17	3.9524	16	20
ESR (mm/h)	1	14.49	14.00	7.8033	9.00	18.00
	2	29.68	30.00	11.9021	18.75	38.00
WBC (×10^9^/L)	1	8.58	8.20	3.4870	6.20	10.50
	2	7.01	6.27	2.9027	5.19	8.42
ANC (×10^9^/L)	1	3.96	3.22	3.4818	1.96	4.73
	2	3.55	3.04	2.1832	2.37	3.81
ALC (×10^9^/L)	1	3.80	3.34	2.0401	2.32	4.92
	2	2.79	2.38	1.4395	1.76	3.13
AMC (×10^9^/L)	1	0.68	0.60	0.3626	0.45	0.82
	2	0.54	0.47	0.2232	0.39	0.68
AEC (×10^9^/L)	1	0.19	0.12	0.2379	0.05	0.23
	2	0.10	0.09	0.0928	0.03	0.15
ABC (×10^9^/L)	1	0.08	0.03	0.5955	0.02	0.05
	2	0.03	0.03	0.0175	0.02	0.04
RBC (×10^12^/L)	1	4.39	4.50	0.6309	4.12	4.80
	2	4.12	4.24	0.6528	3.72	4.63
HGB (×10^9^/L)	1	118	120	15.9971	109	130
	2	113	114	17.7516	102	126
PLT (×10^9^/L)	1	29	22	23.3204	10	43
	2	24	17	23.6401	7	33
MPV (fL)	1	11.30	10.90	2.8088	9.50	12.50
	2	11.19	10.85	3.1913	8.97	12.40
PCT (%)	1	0.07	0.03	0.5024	0.01	0.06
	2	0.08	0.02	0.3130	0.00	0.05
PDW (ratio)	1	16.62	16.70	5.1511	13.65	18.10
	2	17.18	17.05	5.6902	13.78	18.78
Folic acid (nmol/L)	1	38.78	25.21	45.7185	13.28	46.23
	2	51.45	23.06	55.9339	13.53	74.56
Vit B12 (pmol/L)	1	221.82	170.84	232.6191	17.41	369.94
	2	145.81	25.54	194.4035	15.41	236.46
Serum ferritin (ng/mL)	1	217.89	100.23	238.0749	39.52	374.32
	2	377.26	354.25	303.1275	150.37	502.23
EPO (mIU/mL)	1	31.24	13.08	61.3352	8.24	27.62
	2	51.95	17.92	88.9642	10.56	48.96
CD4^+^ (%)	1	35.3	35.2	9.0512	30.0	41.2
	2	36.0	37.7	8.3497	30.8	41.0
CD8^+^ (%)	1	29.4	27.8	9.3721	23.2	33.1
	2	27.3	27.3	5.8100	24.3	30.3
CD4^+^/CD8^+^	1	1.3	1.2	0.5168	1.0	1.6
	2	1.4	1.4	0.5333	1.1	1.7
T cell (%)	1	66.3	67.6	9.7034	60.0	73.3
	2	70.9	70.5	7.2010	67.0	74.9
B cell (%)	1	24.0	22.7	8.2895	18.0	29.2
	2	17.3	17.0	5.0303	13.5	20.9
NK cell (%)	1	8.1	6.3	6.2766	4.0	10.0
	2	8.3	6.7	5.1172	5.4	10.0
T + B + NK cell (%)	1	98.5	98.6	2.4579	97.6	99.3
	2	96.6	97.6	2.9452	95.6	98.7
C3 (g/L)	1	0.85	0.82	0.1854	0.72	0.95
	2	0.67	0.66	0.2097	0.58	0.78
C4 (g/L)	1	0.19	0.17	0.0641	0.14	0.22
	2	0.12	0.13	0.0555	0.09	0.17
IgG (g/L)	1	19.3	19.3	9.4037	10.6	27.3
	2	25.3	23.5	8.7728	20.5	29.9
IgM (g/L)	1	1.01	0.97	0.4288	0.70	1.24
	2	1.42	1.39	0.5938	0.93	1.71
IgA (g/L)	1	0.95	0.88	0.5382	0.53	1.31
	2	1.17	1.01	0.4341	0.89	1.44
IgE (IU/mL)	1	88	42	144.1075	16	97
	2	192	114	205.6359	56	284
CRP (mg/L)	1	8.90	5.00	10.2497	5.00	9.30
	2	8.54	5.72	8.2887	3.37	12.29
IL‐6 (ng/mL)	1	5.71	3.63	6.6737	1.50	7.29
	2	4.92	3.38	3.6106	1.94	7.22
25 (OH) D (nmol/L)	1	61.54	53.78	32.7872	37.40	78.24
	2	47.38	48.64	18.3659	37.56	59.40
TgAb (IU/mL)	1	273.5	210.0	287.1890	17.2	458.0
	2	302.9	276.6	220.7617	143.3	433.3
TPOAb (IU/mL)	1	77.2	76.1	65.2696	14.6	119.9
	2	103.7	86.3	99.3711	45.5	132.8
G‐MK (%)	1	27	27	16.4614	11	43
	2	30	34	15.7824	14	44
PP‐MK (%)	1	6	5	5.3771	2	8
	2	7	5	7.2710	2	8
MN‐MK (%)	1	3	1	13.6334	0	3
	2	3	2	3.5309	1	4
MK‐count (%)	1	204	131	260.3870	70	257
	2	170	128	165.7103	56	213

*Note:* 1 is the pITP group, 2 is the CTD‐TP group. Dry bleeding refers to skin petechiae and ecchymoses. Wet bleeding includes oral mucosal bleeding, gingival bleeding, epistaxis, gastrointestinal bleeding, hematuria, heavy menstrual flow, and cerebral hemorrhage.

Abbreviations: G‐MK, bone marrow megakaryocyte count; MK‐count, bone marrow granular megakaryocytes; MN‐MK, bare nuclei of mature megakaryocytes in bone marrow; PP‐MK, bone marrow megakaryocytes producing platelets; TgAb, antithyroglobulin antibody; TPOAb, thyroid peroxidase antibodies.

**TABLE 3:** Description of baseline count data.

Attribute	Type	Group	
		1	2
Sex (%)	Male	57.9	27.5
	Female	42.1	72.5
Prodromic infection (%)	Positive	45.2	48.7
	Negative	54.8	51.3
Dry bleeding (%)	Positive	77.2	95.0
	Negative	22.8	5.0
Wet bleeding (%)	Positive	36.7	50.0
	Negative	63.3	50.0
ANA (%)	Positive	48.9	92.5
	Negative	51.5	7.5
PAIg (%)	Positive	33.5	34.5
	Negative	66.5	65.5

*Note:* 1 is the pITP group, 2 is the CTD‐TP group.

### 3.2 Variable Screening

Using MI for feature selection, the final variables included in the model are erythrocyte sedimentation rate (ESR), age, complement C3 (C3), complement C4 (C4), weight, immunoglobulin A (IgA), height, immunoglobulin G (IgG), total T + B + NK cell percentage (T + B + NK%), immunoglobulin M (IgM), immunoglobulin E (IgE), bone marrow platelet‐derived megakaryocytes (PP‐MK), antinuclear antibodies (ANA), antithyroid globulin antibodies (TgAb), total B cell percentage (B%), 25 hydroxyvitamin D (25 (OH) D). The lymphocyte count (LY), interleukin‐6 (IL‐6), basophil count (BASO), and platelet hematocrit (PCT) are shown in Figure 2. ESR is the most important feature, with an MI score of 0.0924, far exceeding other features, indicating that ESR has strong discriminative power in distinguishing between the two types of samples. Age (0.0692) ranks second, reflecting the importance of age factors. Complement system indicators C3 and C4 (0.0650 and 0.0642) followed closely behind. Physiological indicators such as weight, immunoglobulin (IgA, IgG), and height also showed good discriminatory ability. It is worth noting that immune‐related indicators (T + B + NK cell percentage, various immunoglobulins) occupy an important position in feature ranking. Overall, the MI scores of the first 20 features showed a decreasing trend, providing a feature selection basis for subsequent model construction.

**FIGURE 2:**
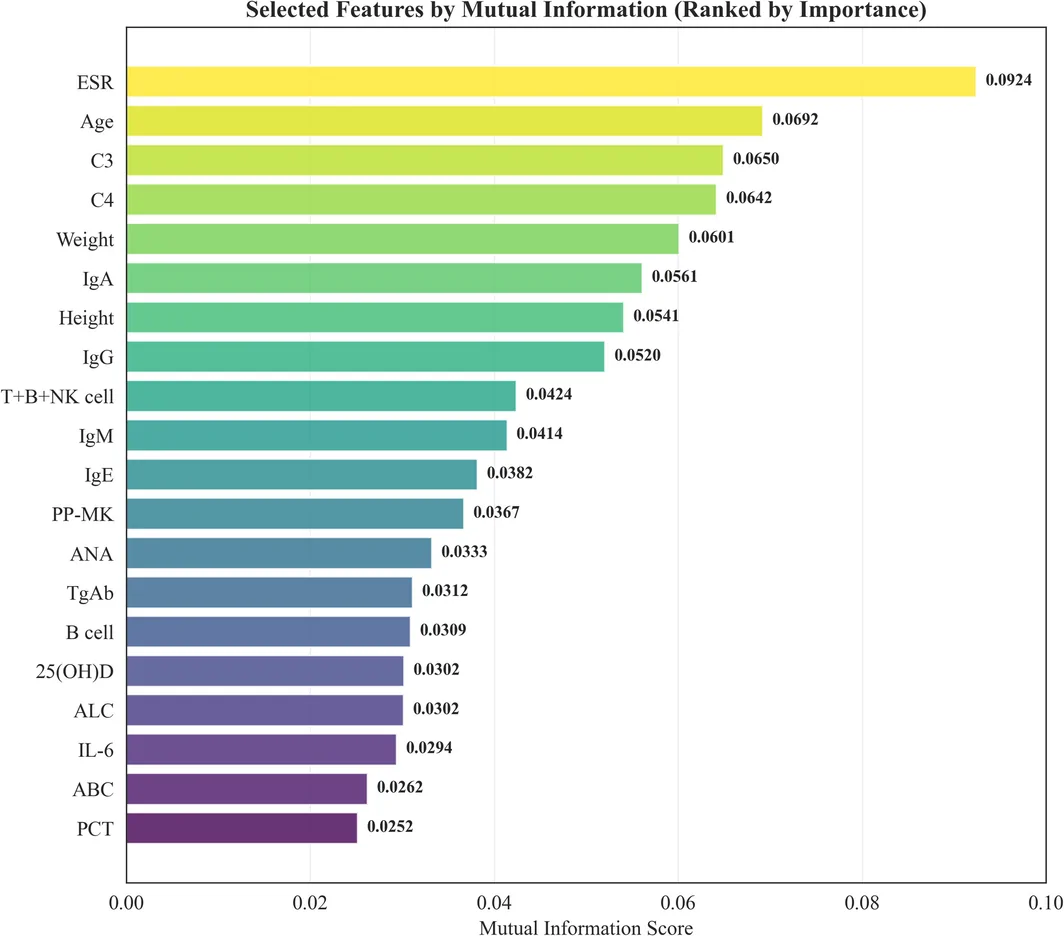
Mutual information feature selection.

### 3.3 Performance Comparison and Analysis of Prediction Models

From the comparative analysis of model performance, as shown in Table 4 and Figure 3, the 6 ML models perform well in all four evaluation indicators, with accuracy generally ranging from 87% to 95%. BPNN performs the best, ranking first in all metrics with an accuracy of 94.87% and an F1 score of 95.04%. LR closely follows, showing stable and balanced performance. SVM and XGBoost have similar performance, with completely consistent accuracy and recall (93.16%). RF performs outstandingly in accuracy (92.92%), while GBDT, although slightly lower in overall performance, still remains within an acceptable range. It is worth noting that the accuracy, recall, and F1 score of all models are very close, indicating that the models can balance false positives and false negatives well on this dataset and have good generalization ability and stability. The confusion matrix of each model is shown in Figure 4. The BPNN ROC curve and Precision recall curve are shown in Figure 5, with AUC values of 0.9738 and 0.7871, respectively. The loss curve is shown in Figure 6, with a smooth decrease indicating normal training and convergence to the horizontal line indicating sufficient model training.

**TABLE 4:** Performance of different machine learning models in the test set.

Model	Accuracy (%)	Precision (%)	Recall (%)	F1_Score (95% CI)
BPNN	94.87 (90.85–98.89)	95.33 (91.42–99.24)	94.87 (90.85–98.89)	0.95 (0.91–0.99)
LR	94.02 (89.95–98.09)	94.82 (90.87–98.77)	94.02 (89.95–98.09)	0.94 (0.90–0.98)
XGBoost	93.16 (88.97–97.35)	92.63 (88.32–96.94)	93.16 (88.97–97.35)	0.92 (0.88–0.96)
SVM	93.16 (88.97–97.35)	93.16 (88.97–97.35)	93.16 (88.97–97.35)	0.93 (0.89–0.97)
RF	92.31 (87.99–96.63)	92.91 (88.63–97.19)	92.31 (87.99–96.63)	0.90 (0.86–0.94)
GBDT	89.74 (84.94–94.54)	87.60 (82.49–92.71)	89.74 (84.94–94.54)	0.88 (0.83–0.93)

**FIGURE 3:**
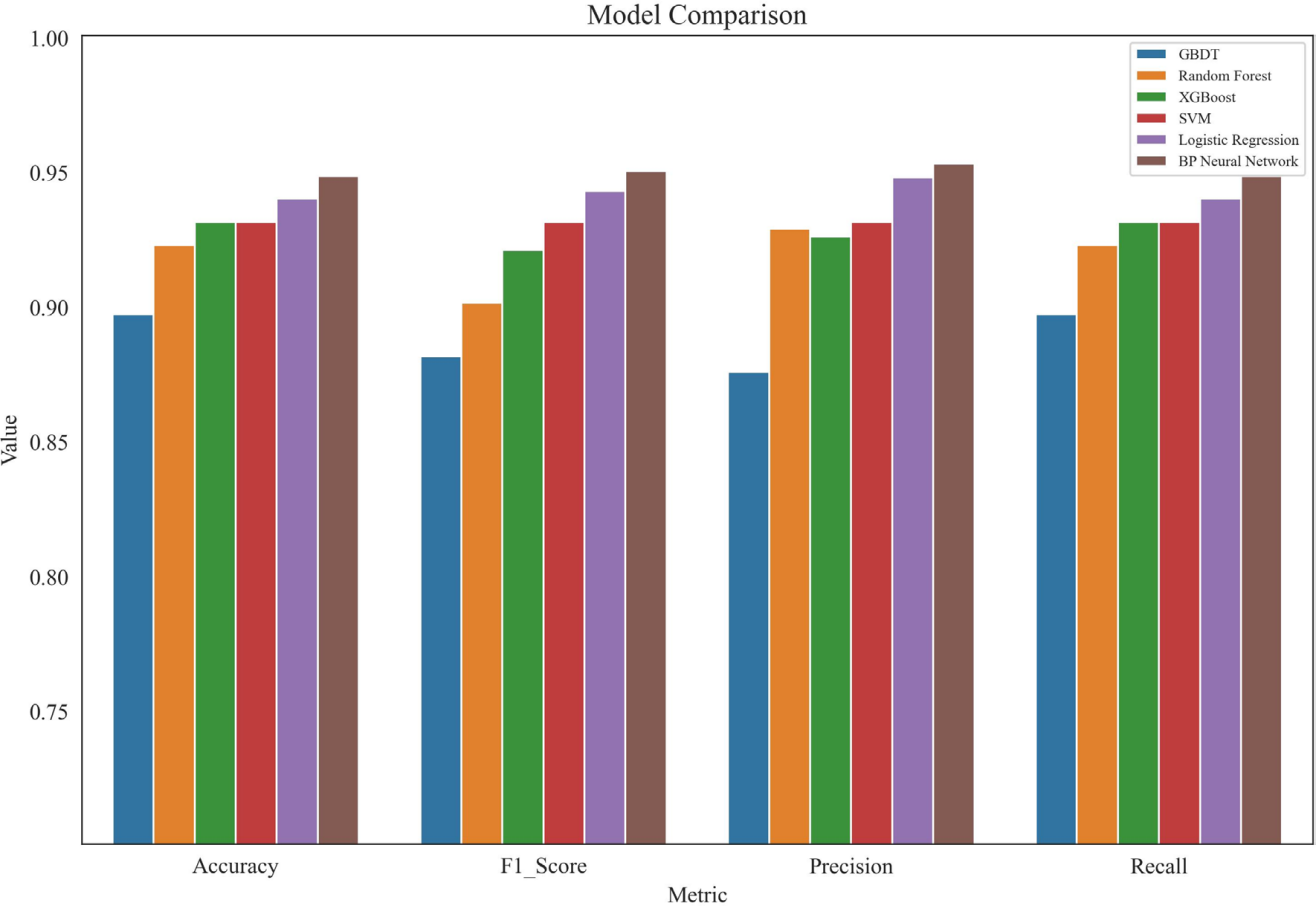
Comparative analysis of models.

**FIGURE 4:**
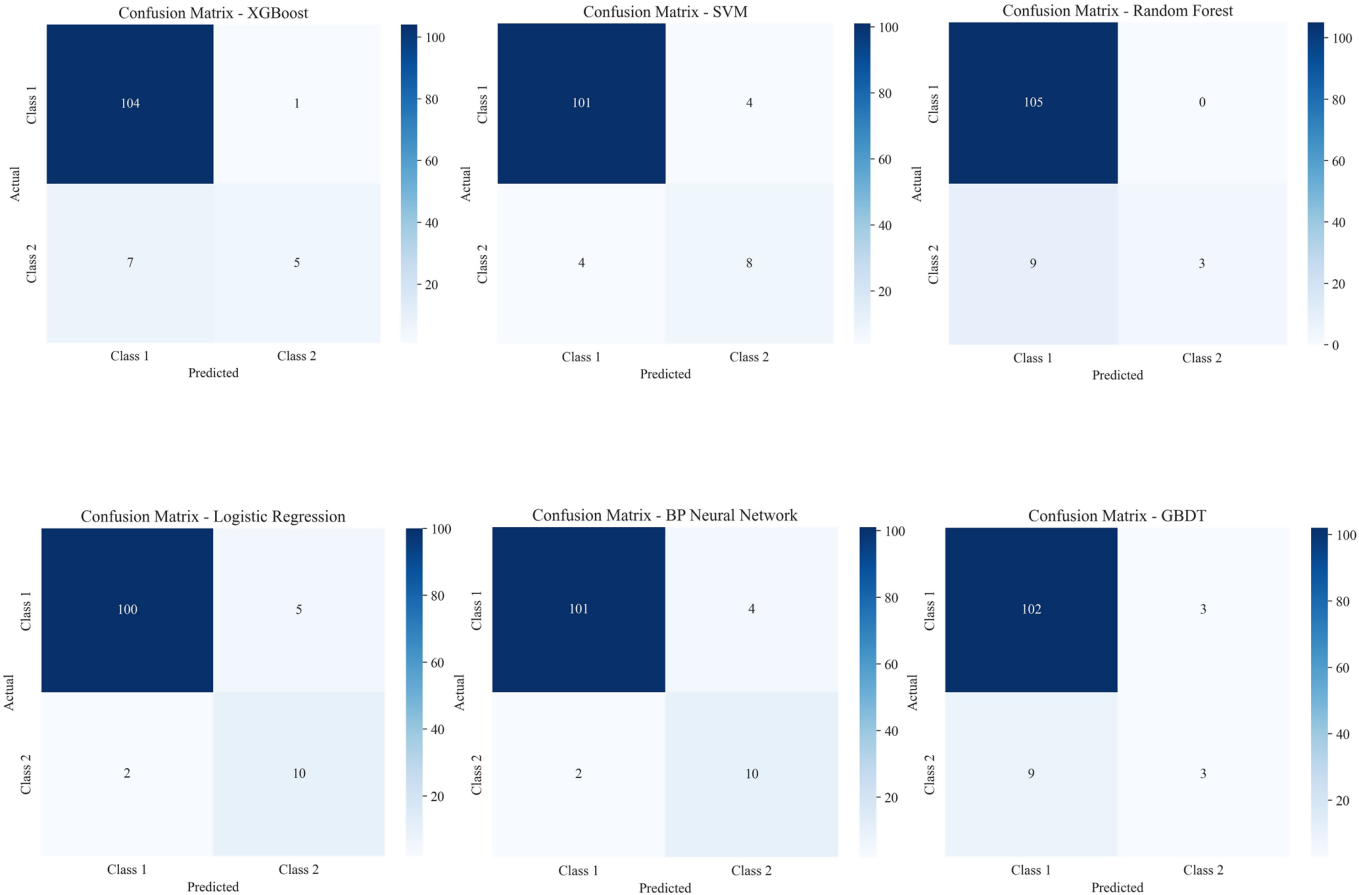
Confusion matrix of each model.

**FIGURE 5:**
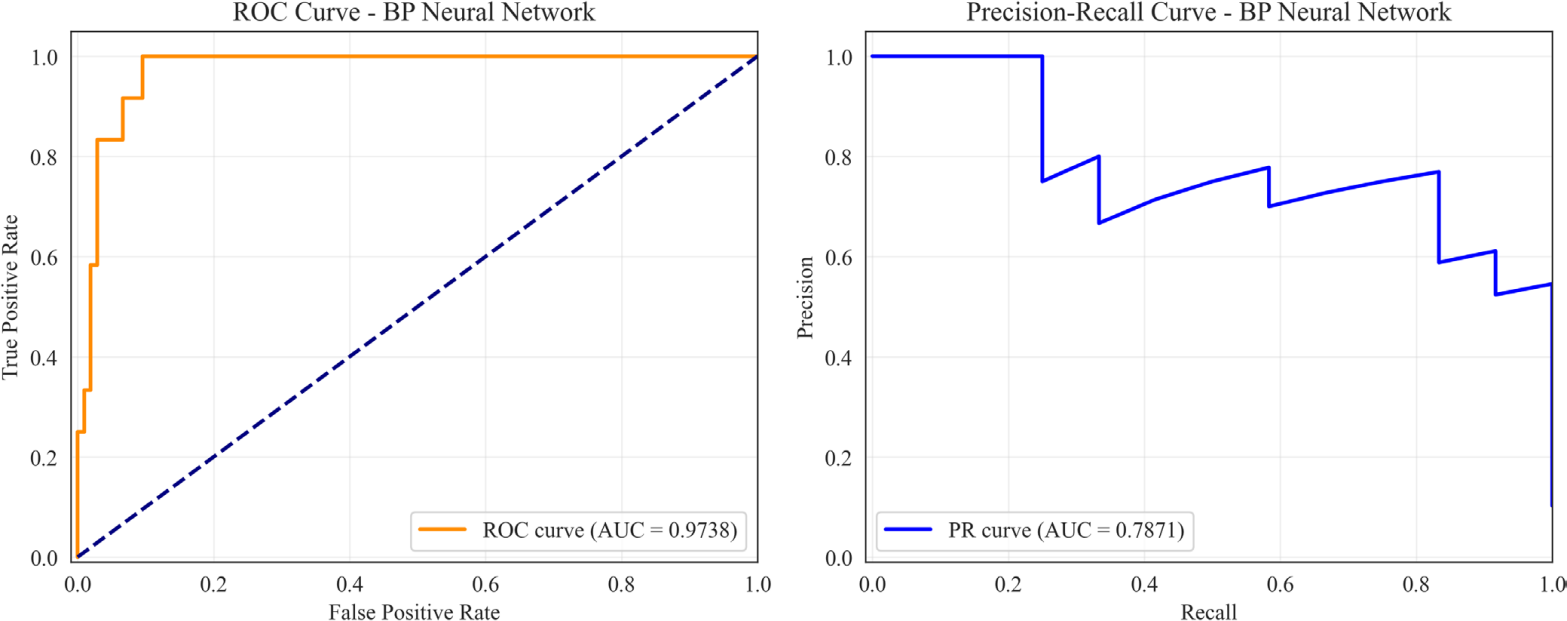
ROC curve and exact recall curve of BP neural network model.

**FIGURE 6:**
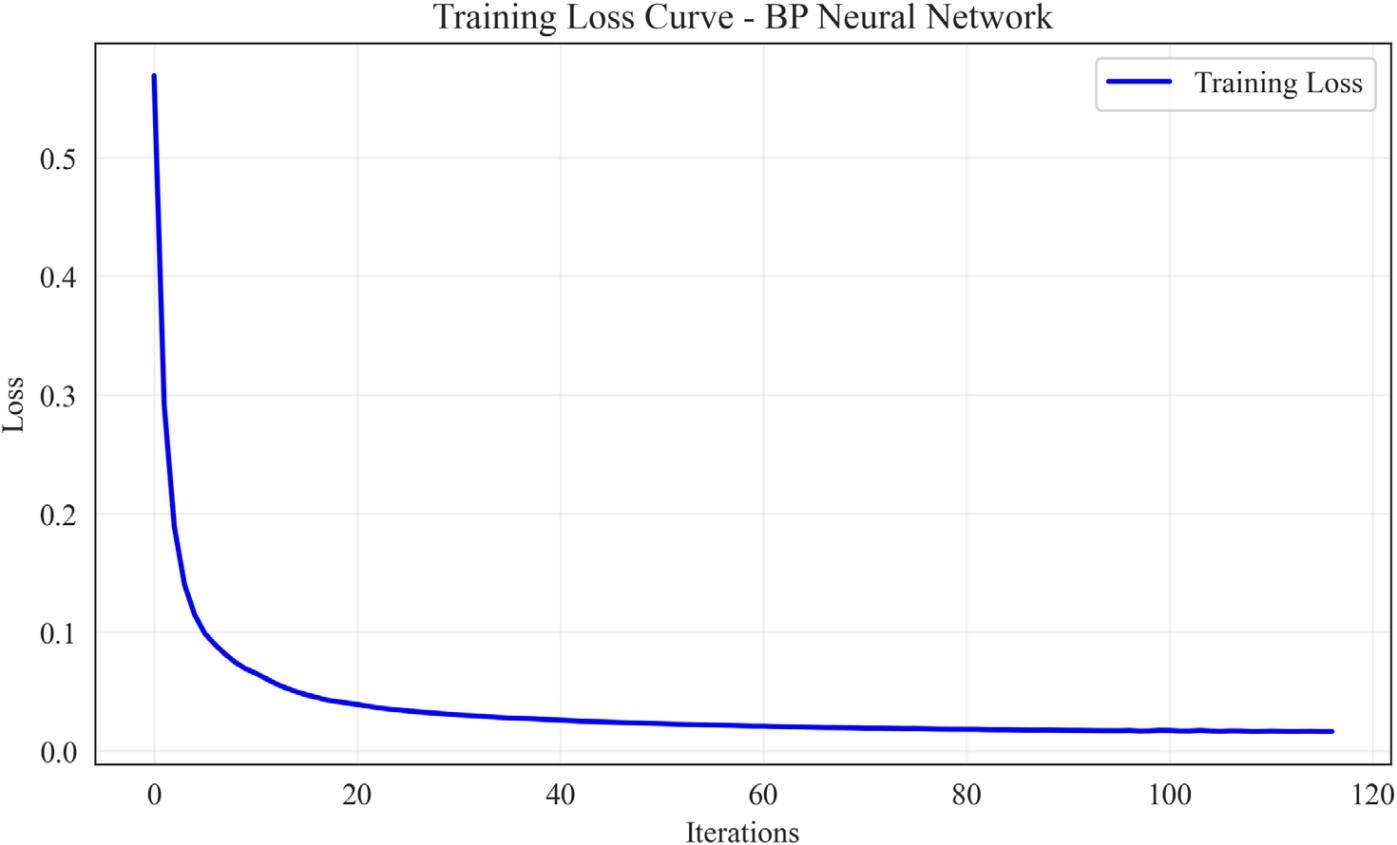
Loss curve of BP neural network model.

### 3.4 SHAP Value Analysis and Important Feature Recognition of BP Neural Network Model

Figure 7 depicts the SHAP bee colony plot of the final BP neural network model and the average impact of SHAP values on the output amplitude of the classifier. These two categories are divided by a hyperplane, with CTD‐TP cases on the right side of the hyperplane and pITP cases on the left side of the hyperplane. In addition, higher and lower values are represented in red and blue, respectively. In addition, these tags are sorted in descending order of importance. The best attribute is still at the top. It can also be clearly seen from the figure that CTD‐TP patients are older than pITP patients, with increased erythrocyte sedimentation rate and IgM levels, and decreased levels of C3, C4, B cells, and T + B + NK cells. According to SHAP, age, ESR, ANA, IgM, C3, T + B + NK%, B cells, body weight, C4, and ALC are necessary indicators for distinguishing CTD‐TP from pITP.

**FIGURE 7:**
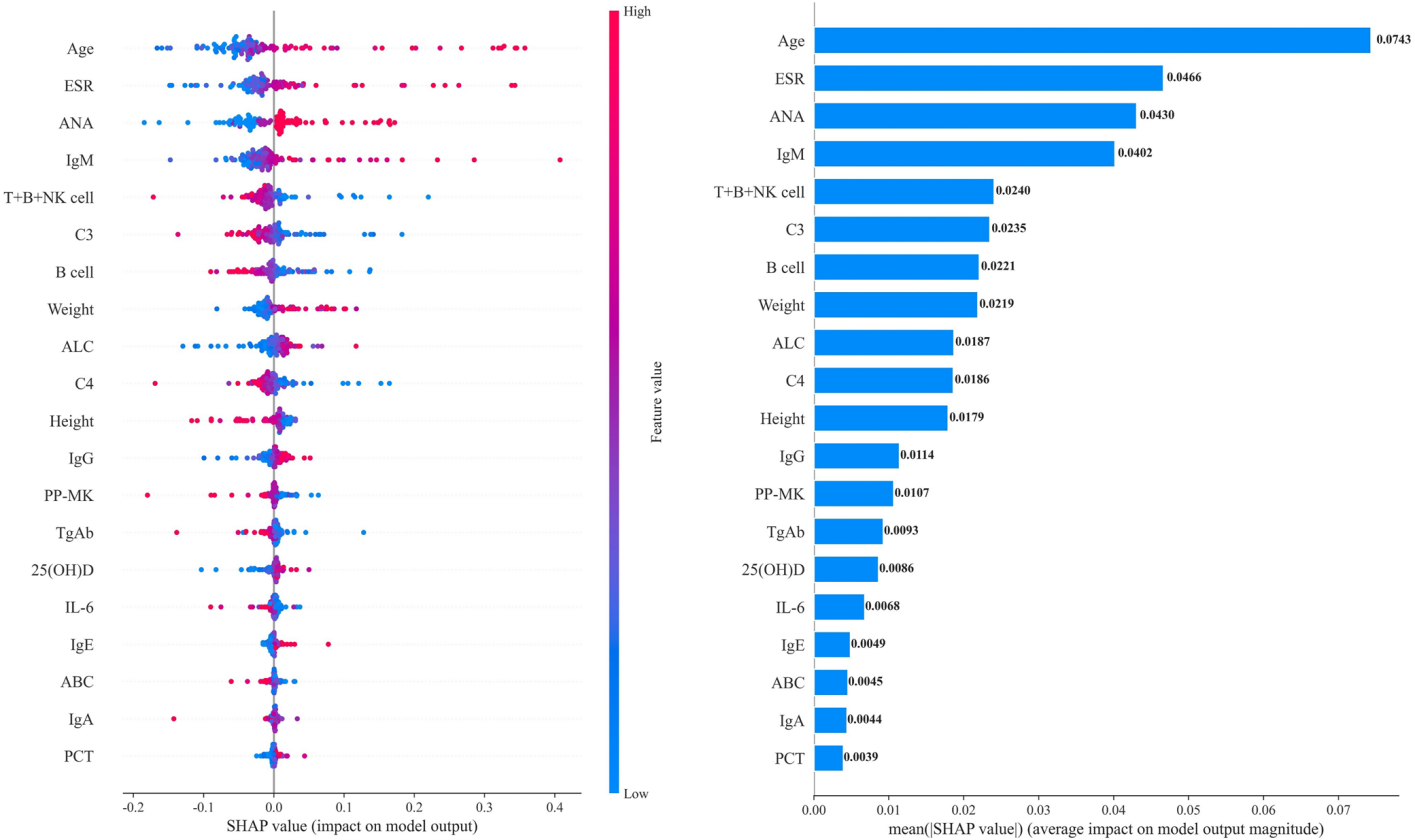
Bee swarm plot using SHAP and Bar Chart using SHAP.

## 4 Discussion

ITP is a common hemorrhagic disease characterized by bleeding and reduced peripheral blood PLT. The causes of PLT reduction are excessive destruction of PLT mediated by humoral and cellular immunity, as well as insufficient PLT production due to abnormal quantity and quality of megakaryocytes [19]. CTD is a chronic autoimmune disease that involves multiple systems and organs. It can present with a specific clinical symptom or systemic damage as the initial manifestation, and has no specificity in the early stages, with hematological involvement being more common [20]. Therefore, CTD patients with PLT reduction as the initial symptom are easily misdiagnosed as pITP in clinical practice and need to be identified early. In recent years, ML has relied on mathematical algorithms to deeply mine the associations between variables and diseases, demonstrating high efficiency in processing complex medical data [21]. This study is the first to construct early differential diagnosis models for pITP and CTD‐TP based on six different ML algorithms. The predictive performance and clinical practicality of each model are comprehensively compared, and multiple evaluation tools are used to comprehensively evaluate the accuracy, stability, and generalization of the models. Finally, a model based on BP neural network is selected. At the same time, the importance of different variables in the model was ranked and analyzed. The results showed that age, erythrocyte sedimentation rate, antinuclear antibodies, IgM, Complement C3, T + B + NK%, B cells, body weight, complement C4, and lymphocyte count have high discriminatory value.

In the BP neural network model established in this study, the feature importance evaluation results showed that the top three variables that contributed the most to the model were age, ESR, antinuclear antibodies, similar to previous studies, indicate that children who are more susceptible to CTD are more likely to be in puberty and often have positive anti nuclear antibodies. The presence of autoantibodies is used for the diagnosis of CTD The increase in antibody titers and inflammatory markers (ESR) is correlated with the severity and activity of CTD, and has significant biomarker value in monitoring disease progression and treatment response [22, 23]. In addition, IgM, complement C3, T + B + NK%, B cells, body weight, complement C4, and lymphocyte count have good clinical application value in distinguishing between the two diseases. According to literature reports, complement C3 and C4 in CTD serum were significantly reduced, indicating that C3 and C4 were consumed and the complement system was activated. The degree and changes of serum complement C3 and C4 can be used to clarify the disease activity of CTD [24], which is consistent with our study. Our research results showed that children with CTD had reduced lymphocyte counts, T + B + NK%, decreased B cells, and increased IgM levels, indicating that autoimmune factors play an important role in the pathogenesis of CTD‐TP. Relevant research results suggest that the reason for CTD‐induced PLT reduction may be due to lymphocyte dysfunction, enhanced B lymphocyte function, and immune dysfunction, leading to the production of various autoantibodies, which is consistent with the results of this study [8, 25]. In this study, it was found that some children with platelet abnormalities as the initial diagnosis of CTD may not have typical clinical symptoms, have not undergone relevant immunological examinations, or have diagnostic criteria that do not meet the diagnostic criteria for diagnosing CTD after completing relevant immunological examinations. After a period of time, other typical symptoms gradually appear or the abnormal examination results reach the diagnostic criteria before the diagnosis can be confirmed, resulting in a decrease in the early diagnosis rate of CTD with platelet abnormalities as the initial diagnosis and a prolongation of the time from onset to diagnosis [26]. Our model provides relatively clear conclusions and has certain reference value for early clinical identification.

## 5 Conclusion

In summary, this article establishes and validates a BP neural network model based on multiple independent risk factors through simple clinical features, with an accuracy of 94.87% and an F1 score of 0.95. The results show that age, erythrocyte sedimentation rate, antinuclear antibodies, IgM, Complement C3, T + B + NK%, B cells, body weight, complement C4, and lymphocyte count have good clinical value in the differential diagnosis of ITP and CTD‐TP. In clinical practice, early identification and monitoring of newly diagnosed children with thrombocytopenia can be based on the importance of variables. When making preliminary judgments on their basic clinical characteristics such as age, immunological indicators should be comprehensively considered. However, if there is a lack of typical manifestations of CTD in the early stages of the disease, no evidence of other systemic damage, and only positive autoantibodies, early identification is difficult. At this time, long‐term dynamic monitoring of the autoantibody spectrum and immunological indicators is also necessary, which is of great significance for guiding clinical decision‐making and early intervention.

This study has some limitations. First, it is a single‐center retrospective study with a limited sample size, which might restrict how well the results can be applied to broader populations and different clinical settings. Second, there is an inherent class imbalance between the pITP group and the CTD‐TP group—while this does reflect the real epidemiological distribution of the disease (e.g., secondary ITP accounts for about 40% in some Asian populations), it could introduce bias in model training and affect the performance of some algorithms. Third, the lack of an external independent dataset for validation limits the robustness and generalizability of the model. Fourth, the retrospective design restricts standardized data collection, which might introduce information bias. Future studies should involve large‐sample, multicenter prospective research and include external validation cohorts to further confirm the clinical utility of this model.

## Author Contributions

Furong Kang, Mei Yan, Yu Liu: experimental design, research guidance, article writing. Furong Kang, Yingbin Yue, Xuemei Wang: data statistical analysis, data collection and organization, paper review.

## Funding

This study was supported by the Basic Research on Major Pediatric Diseases, Specialized Disease Cohort Study, and Early Warning Model Construction (program number: 2024B03038‐1), and the Etiology Exploration and Prognostic Prediction Cohort Study of Pediatric Immune Thrombocytopenia, Its Application Promotion (program number: TSYC202301A002), and the Impact of Prechemotherapy Serum Metabolomics and Nutritional Indices on the Prognosis of Pediatric Acute Lymphoblastic Leukemia (program number: 2024D01C169).

## Ethics Statement

Approval was obtained from the ethics committee of the First Affiliated Hospital of Xinjiang Medical University (Approval number 241008‐01). The procedures used in this study adhere to the tenets of the Declaration of Helsinki.

## Consent

Informed consent was obtained from all individual participants included in the study.

## Conflicts of Interest

The authors declare no conflicts of interest.

## Data Availability

The authors have nothing to report.
